# Circulating organokines in coronary artery disease and metabolic syndrome: FABP4, adiponectin, irisin, FSTL1

**DOI:** 10.17305/bb.2025.13188

**Published:** 2025-11-04

**Authors:** Meltem Uyaner Kan, İbrahim Kilinc, Hakan Akilli, Hasan Huseyin Bilgic

**Affiliations:** 1 Department of Medical Biochemistry, Necmettin Erbakan University, Konya, Türkiye; 2 Department of Cardiology, Necmettin Erbakan University, Konya, Türkiye; 3 Department of Aeronautical Engineering, Necmettin Erbakan University, Konya, Türkiye

**Keywords:** Metabolic syndrome, coronary artery disease, fatty acid-binding proteins, adiponectin, follistatin-related proteins, irisin

## Abstract

Cardiovascular disorders are closely linked to metabolic syndrome and remain a leading cause of mortality worldwide, despite advances in early detection and treatment. Adipokines, cardiokines, and myokines play critical roles in maintaining systemic metabolic homeostasis. In this study, we measured serum levels of fatty acid binding protein 4 (FABP4), follistatin-like 1 (FSTL1), irisin, and adiponectin in 243 male patients undergoing elective coronary angiography. We investigated the associations of these biomarkers with coronary artery disease (CAD) and their correlation with metabolic syndrome status. FSTL1 levels were predicted using a particle swarm optimization-enhanced adaptive neuro-fuzzy inference system (PSO-ANFIS) based on artificial intelligence. Patients with CAD exhibited significantly lower FABP4 levels (*P* < 0.0001), and low FABP4 levels emerged as an independent predictor of CAD in logistic regression analysis (odds ratio 0.903, 95% CI 0.825–0.987, *P* ═ 0.025). The combination of adiponectin, FSTL1, and irisin as a biomarker strategy demonstrated high sensitivity and specificity for diagnosing metabolic syndrome (AUC = 0.92, 95% CI 0.88–0.96). Both FSTL1 and adiponectin independently correlated with metabolic syndrome (*P* < 0.001, odds ratio 1.039, 95% CI 1.025–1.054; *P* < 0.001, odds ratio 0.979, 95% CI 0.971–0.988, respectively). The prediction of FSTL1 levels using PSO-ANFIS supports the concept of harmonization among metabolic messengers. These findings underscore the potential of FABP4 and FSTL1 as valuable biomarkers for diagnosing metabolic and cardiovascular diseases, thereby facilitating personalized interventions targeting organokine pathways.

## Introduction

Adipose tissue, skeletal muscle, and the heart function as active endocrine organs, synthesizing and secreting bioactive molecules known as adipokines, myokines, and cardiokines under both physiological and pathological conditions. Given that atherosclerosis is recognized as a chronic inflammatory disease, there has been considerable interest in inflammation markers and related studies. These molecules and their interactions have been implicated in inflammatory and oxidative damage, obesity, and obesity-related cardiovascular diseases [1].

Transcriptomic analyses of human adipose tissue have been crucial in shaping the concept of adipokines. Adiponectin, notable for its adipocyte-specific secretion and distinct collagen-like and nectin-like structural features [2], serves as a key adipokine with metabolic functions, including the enhancement of glucose and lipid homeostasis, as well as the attenuation of oxidative stress and inflammation [3].

Adipokines facilitate interactions between adipose tissue and peripheral organs, including the heart. Fatty acid-binding proteins (FABPs) are a group of cytosolic proteins that play significant roles in lipid transport and the coordination of inflammatory and metabolic pathways. Adipocyte-FABP (A-FABP, also known as aP2 or FABP4) is a cytosolic protein predominantly found in adipocytes, but also expressed in macrophages and endothelial cells. Elevated serum FABP4 concentrations are associated with obesity, insulin resistance, hypertension, inflammation, atherosclerosis, and metabolic syndrome, thus positioning FABP4 as a potential independent biomarker for metabolic and cardiovascular diseases [4].

Skeletal muscle constitutes approximately 40% of body weight and is the largest organ in non-obese individuals. Numerous myokines produced and secreted by skeletal muscle have been identified. Irisin, a notable myokine, is derived from a Type I membrane precursor protein known as fibronectin Type III domain-containing protein 5. After proteolytic degradation, irisin is released into circulation. Recognized as an exerkine, irisin has been shown to reduce insulin resistance in muscle [5] and is believed to contribute to the beneficial effects of exercise on metabolism by promoting the conversion of white adipose tissue into brown adipose tissue.

Proteins secreted by cardiac cells, studied through transcription, protein, and secretome expression analyses in cardiac tissues, are referred to as cardiokines [6]. Cardiokines (or cardiomyokines) are peptides and proteins secreted by cardiac cells—including myocytes, fibroblasts, endothelial cells, and vascular cells—under both physiological and pathological conditions, exerting autocrine, paracrine, and potentially endocrine effects. These proteins, such as follistatin-like 1 (FSTL1), are involved in regulating processes including inflammation, fibroblast activation, cardiomyocyte hypertrophy, and cardiac remodeling [7].

Cardiovascular diseases encompass a range of disorders affecting the heart and blood vessels and remain a leading cause of mortality globally [8]. Metabolic syndrome is characterized by the coexistence of multiple risk factors that contribute to the development of cardiovascular diseases. Among the various cardiovascular conditions, coronary artery disease (CAD) represents a significant health burden, characterized by the accumulation of atherosclerotic plaques—either obstructive or non-obstructive—within the epicardial coronary arteries [9]. Asymptomatic atherosclerotic CAD is prevalent in the general population [10]. In this context, identifying metabolic messengers capable of predicting, preventing, or reducing mortality before the onset of cardiac damage is crucial. This study aims to investigate the association between circulating organokines and CAD as well as metabolic syndrome.

## Materials and methods

### Study population

**Figure 1.**
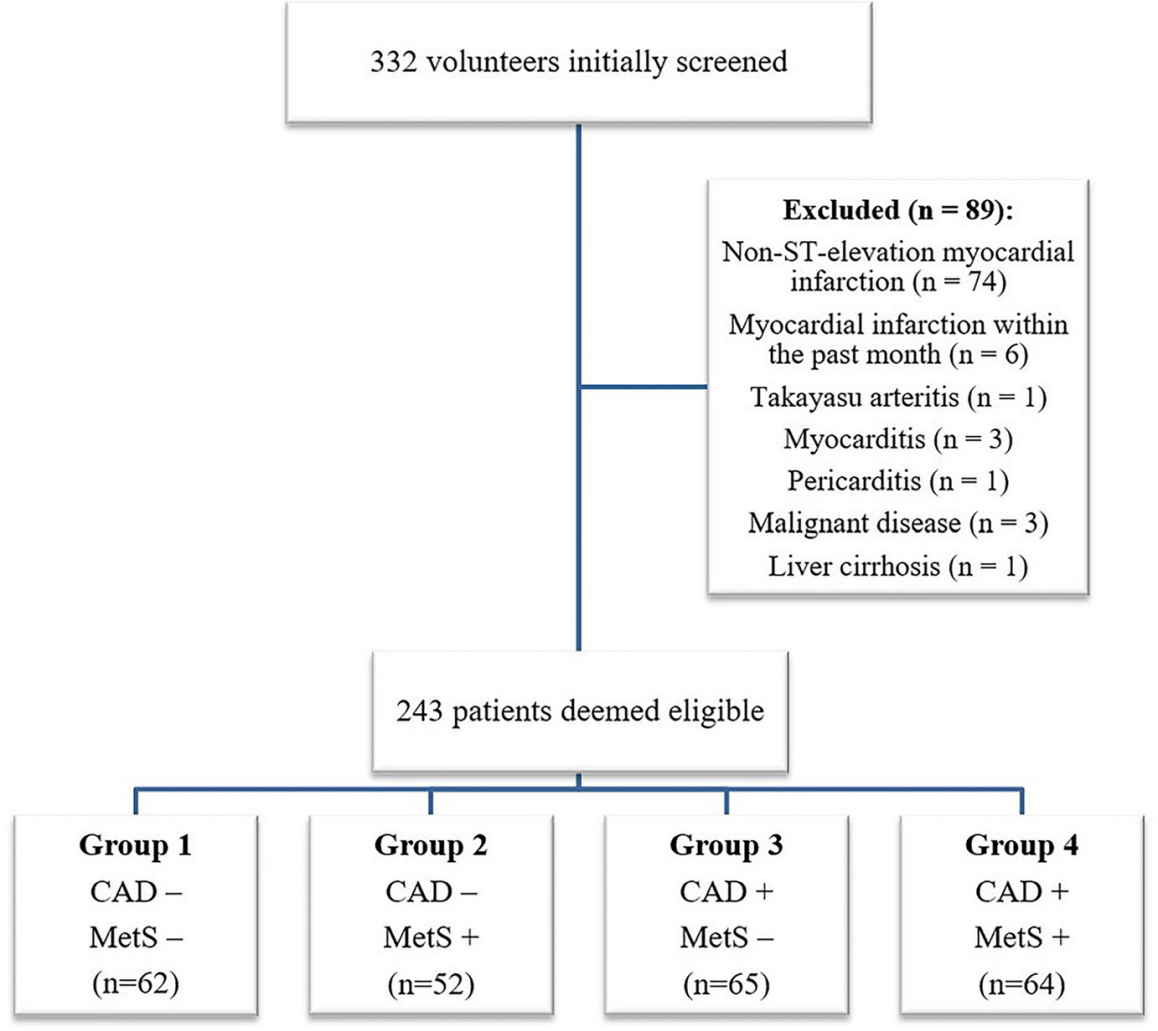
**Classification of patients into study groups.** Abbreviations: MetS: Metabolic syndrome; CAD: Coronary artery disease.

The study was conducted prospectively from October 2023 to May 2024, involving male patients aged 18 years and older who presented to the Coronary Angiography and Cardiac Catheterization Laboratory. Patients with neurological, musculoskeletal, or gastrointestinal diseases, as well as those with thyroid, adrenal, or gonadal hormone disorders, infections, oncological diseases, or acute coronary syndrome were excluded. Based on the presence of metabolic syndrome (as defined by the National Cholesterol Education Program Adult Treatment Panel III [11]) and CAD, patients were classified into four groups (Figure 1). CAD was categorized according to the SYNTAX score, which assesses lesions causing ≥50% stenosis in vessels ≥1.5 mm, with the coronary tree divided into 16 segments based on the AHA classification, as modified in the ARTS I and II trials [12].

### Biochemical measurements

All venous blood samples were collected from fasting participants in the morning, allowed to clot for 30 min, and then centrifuged at 1500 *g* for 15 min. Serum aliquots were stored at −80 ^∘^C until analysis to minimize pre-analytical variability. Human sandwich enzyme-linked immunosorbent assay (ELISA) kits from ELK Biotechnology were utilized to measure serum concentrations of adiponectin, FABP4, FSTL1, and irisin (intra-assay CV < 8%, inter-assay CV < 10%). The detection limits were 0.16 ng/mL for adiponectin, 31.25 pg/mL for FABP4 and FSTL1, and 15.63 pg/mL for irisin. Additional details regarding biochemical measurements and calibration curves (Figures S1–S12) are provided in the supplementary material.

The Sysmex XN-1000 analyzer was employed to conduct complete blood count tests, while the Premier Hb9210 automatic analyzer was utilized for HbA1c testing. HDL, LDL, CRP, glucose, urea, and creatinine levels were measured using spectrophotometry with the Roche Cobas 8000 c702 analyzer. Furthermore, insulin, high-sensitive cardiac troponin T, and IL-6 levels were assessed using electrochemiluminescence immunoassay on the Roche Cobas 8000 e801 device.

### Ethics approval and reporting guidelines

On June 16, 2023, the Necmettin Erbakan University Ethics Committee approved the study protocol (Approval No: 2023/4389). All participants provided written informed consent, and the study adhered to the Declaration of Helsinki. Additionally, the design of the study complied with the Strengthening the Reporting of Observational Studies in Epidemiology (STROBE) criteria.

### Statistical analysis

Statistical analyses were conducted using GraphPad Prism v10, Jamovi v2.3.28, SPSS v26.0, and RStudio v2025.09.1. The sample size was calculated using G*Power 3.1, determining a requirement of 180 participants distributed evenly across four groups, with a 5% error margin, 80% power, and a medium effect size (0.25). To mitigate potential participant attrition, the sample size was increased by 10%, ensuring a minimum of 50 participants in each group. Data collection concluded once the requisite sample size was achieved.

Descriptive data are presented as medians with interquartile ranges (IQR), means ± standard deviation (SD), counts (*n*), or percentages (%). The Shapiro–Wilk test assessed the normality of data distribution. Categorical variables were compared using Pearson’s chi-square test with Bonferroni correction. Continuous variables were evaluated with non-parametric tests (Mann–Whitney *U* test and Kruskal–Wallis test), except for age, which was analyzed using one-way ANOVA. Post-hoc tests (Dunn’s test, Tukey test, and Bonferroni correction) identified sources of significant differences among three or more groups.

Relationships between adiponectin, FABP4, FSTL-1, irisin, and other variables were examined using Spearman’s correlation test. Binary logistic regression identified independent factors associated with CAD and assessed the effects of parameters on metabolic syndrome while adjusting for confounding variables. Events per variable were evaluated for all multivariable models to ensure sufficient statistical power. Statistical significance was set at *P* < 0.05 for all tests.

### Result prediction with particle swarm optimization-based adaptive neuro-fuzzy inference system

**Figure 2.**
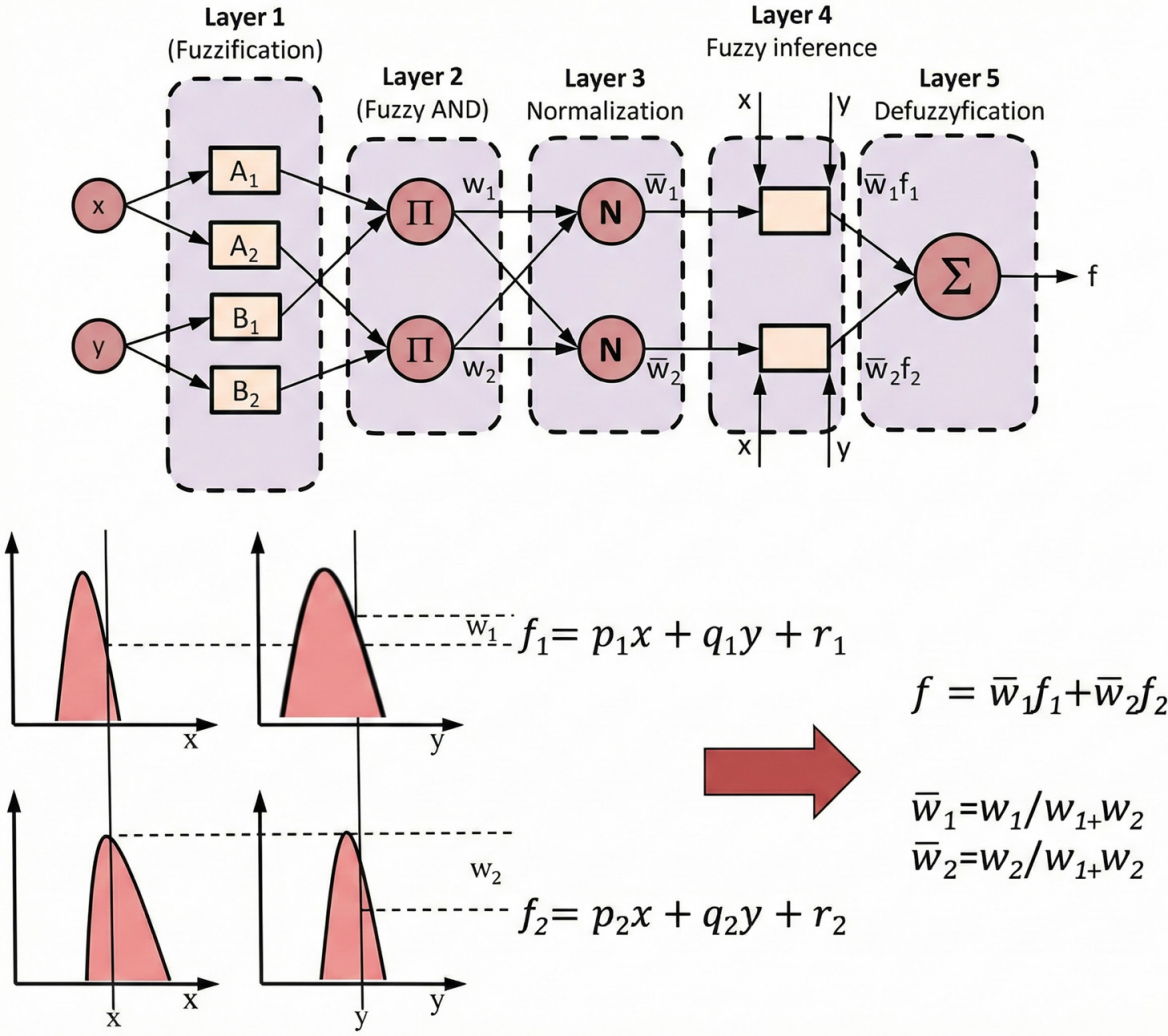
**Background of adaptive neuro-fuzzy inference system.** The operational structure and mathematical background of the adaptive neuro-fuzzy inference system are illustrated.

Prediction models utilizing artificial intelligence methods are increasingly prevalent in the literature [13–15]. One notable approach is the adaptive neuro-fuzzy inference system (ANFIS), which integrates the parallel processing capabilities of artificial neural networks with the expert knowledge-based framework of fuzzy logic [16]. The operational structure and mathematical foundations of ANFIS, initially proposed by Jang in 1993, are illustrated in Figure 2 [17].

A critical determinant of the ANFIS architecture’s success is the selection of membership function types and weights, as well as the establishment of model weights and rule sets. While these selections have traditionally relied on conventional methods, the predictive performance of ANFIS models can be significantly improved through the application of metaheuristic search algorithms. Among these advanced metaheuristic algorithms, particle swarm optimization (PSO) has proven to be an effective tool for modeling nonlinear systems akin to biological processes [18]. Consequently, modeling processes that incorporate PSO-enhanced ANFIS can achieve substantially higher accuracy.

In this study, artificial intelligence analyses were conducted using MATLAB R2024b and Orange Data Mining 3.38.1. The weights and rules for the ANFIS model were determined through the PSO algorithm. The parameters utilized in this study included a maximum of 100 iterations, a population size of 40, an inertia weight of 1, an inertia weight damping rate of 0.99, a personal learning coefficient (c1) of 1, and a global learning coefficient (c2) of 2. Gaussian membership functions were employed, with nine membership functions defined for each input variable. Input values were normalized to the range [0–1]. Following outlier analysis using the Tukey robust statistic, artificial intelligence analysis was conducted on patients with complete data, ensuring the same cohort of 220 patients was utilized across all methods. A fixed random seed was established to ensure reproducibility. To demonstrate the applicability of the proposed approach, the dataset was divided into two subsets: training and testing, with 80% allocated to training and 20% to testing. This division facilitated the assessment of potential overfitting, a common challenge in predictive modeling.

## Results

**Figure 3.**
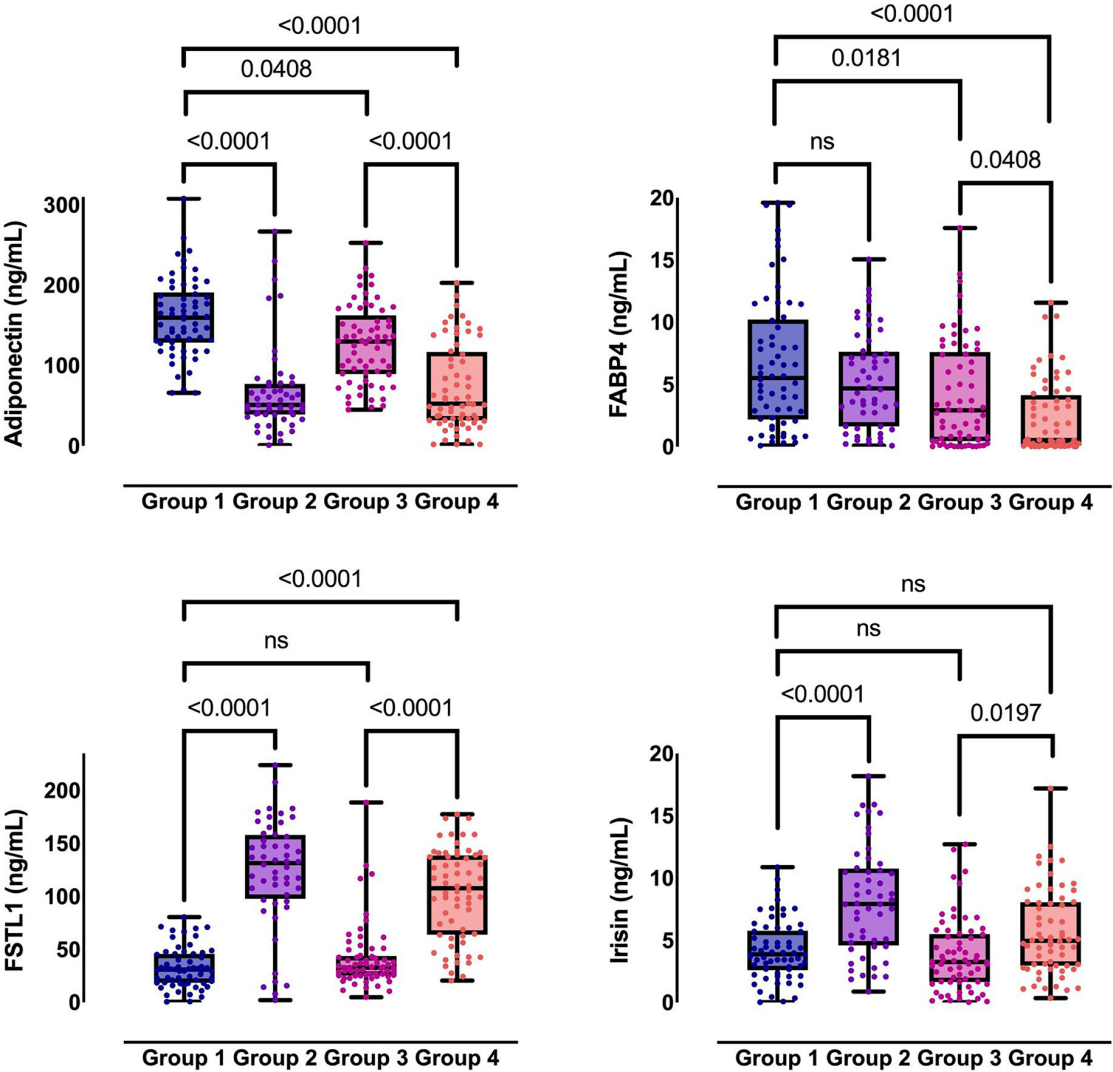
**Levels of adiponectin, FABP4, FSTL1, and irisin in patients stratified by MetS and CAD.** Group 1 (*n* ═ 62): MetS-negative/CAD-negative; Group 2 (*n* ═ 52): MetS-positive only; Group 3 (*n* ═ 65): CAD-positive only; Group 4 (*n* ═ 64): MetS-positive/CAD-positive. Data are presented as medians with IQR, as detailed in Table 1. Pairwise significance markers represent multiplicity-adjusted post-hoc tests (Dunn with Bonferroni). *P* values were adjusted for multiple comparisons across the four biomarkers using the FDR method. Abbreviations: FABP4: Fatty acid-binding protein 4; FSTL1: Follistatin-like 1; MetS: Metabolic syndrome; CAD: Coronary artery disease; *n*: Sample size; IQR: Interquartile range; FDR: False discovery rate; ns: Not significant; *P*: Significance.

The demographic and anthropometric characteristics, clinical features, and biochemical parameters of the study participants are detailed in Table 1. When evaluated collectively across all four groups, the presence of CAD significantly influenced levels of FABP4 and irisin. Within the context of metabolic syndrome, notable differences were observed in the levels of adiponectin, irisin, and FSTL1 (refer to Table 1 and Figure 3). A significant moderate correlation was identified between fasting serum triglyceride levels and both serum FSTL1 (*r* ═ 0.43) and serum irisin levels (*r* ═ 0.423) (*P* < 0.001) across all participants. Conversely, an inverse significant correlation was found between serum adiponectin and FSTL1 levels (*r* ═ –0.672, *P* < 0.0001, Table S1).

In the CAD risk factor model, generated through binary logistic regression analysis and adjusted for confounding variables (including dyslipidemia, defined as total cholesterol ≥200 mg/dL, LDL-C ≥130 mg/dL, triglycerides ≥150 mg/dL, HDL-C <40 mg/dL or the use of lipid-lowering therapy; hypertension; age; smoking; and fasting glucose levels), irisin did not maintain statistical significance as an independent predictor. In contrast, FABP4 remained significantly associated with the presence of CAD (Table 2). According to the ROC analysis, FABP4 exhibited an area under the curve (AUC) of 0.702 for diagnosing CAD. To illustrate the diagnostic performance of FABP4 at varying clinical priorities (higher sensitivity for screening and higher specificity for confirmatory evaluation), two cut-off values determined using Youden’s J index were included: for 6.422 ng/mL, the sensitivity was 80.6%, and for 0.584 ng/mL, the specificity was 92.1% (Table S2).

For the diagnosis of metabolic syndrome, a combined biomarker score was calculated as the logistic regression linear predictor, incorporating adiponectin, irisin, and FSTL1. The predicted probability (combined biomarker score) was derived using Equation (1).


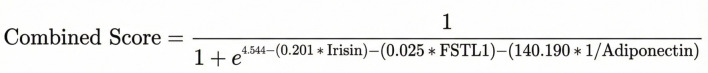
 (1)

**Figure 4.**
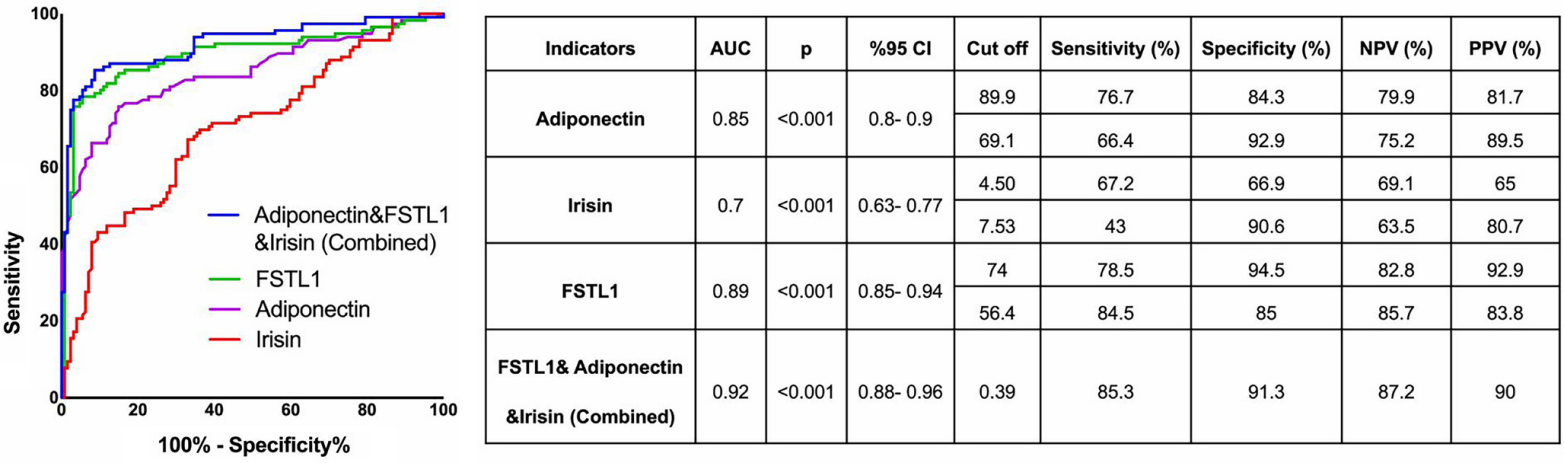
**ROC curves comparing the discriminatory performance of adiponectin, FSTL1, irisin and combined biomarker score in identifying patients with MetS.** Mean AUC values and their 95% confidence intervals were estimated using 200 bootstrap samples. DeLong test comparing ROC curves of FSTL1 and the combined biomarker: Z ═ −2.019, *P* ═ 0.044. Moreover, optimal cut-off points determined via the Youden index, along with corresponding sensitivity and specificity values, are presented. Abbreviations: AUC: Area under the curve; *P*: Significance; CI: Confidence interval; NPV: Negative predictive value; PPV: Positive predictive value.

The calculated cut-off value for the combined biomarker score was 0.389, with an area under the ROC curve (AUC) of 0.92. The appropriate cut-off values and characteristics of adiponectin, irisin, FSTL1, and the combined score for diagnosing metabolic syndrome in our study are presented in Figure 4.

**Table 1:** Clinical and demographic characteristics of the participants

	***n***	**Group 1 (*n* ═ 62)**	**Group 2 (*n* ═ 52)**	**Group 3 (*n* ═ 65)**	**Group 4 (*n* ═ 64)**	***P***
Age	243	56.2±10.6	56.2±12.2	62.7±9.9	62.3±9.2	0.0001^*^
BMI (kg/m^2^)	243	26.1 (24.4–29.7)	30.2 (27.7–34.2)	26.1 (23.5–30)	28.7 (26.4–30.5)	<0.0001^#^
WC (cm)	199	96.5 (90–100)	100 (100–110)	98 (90–105)	103.5 (100–110)	<0.0001^#^
WHtR	199	0.55 (0.52–0.59)	0.60 (0.58–0.64)	0.56 (0.52–0.63)	0.61 (0.58–0.65)	<0.0001^#^
Smoker: (%)	213	37	27.5	32.2	35	0.788^†^
DM: *n*(%)	243	5 (8.1)	29 (55.8)	9 (13.8)	42 (65.6)	<0.0001^†^
Hypertension: *n*(%)	243	20 (32.3)	35 (67.3)	25 (38.5)	45 (70.3)	<0.0001^†^
Statin usage: *n*(%)	243	13 (20.9)	18 (34.6)	28 (43)	46 (71.9)	<0.0001^†^
SYNTAX score	243	0	0	13 (8–21)	15 (9.3–21)	<0.0001^#^
Triglyceride (mg/dL)	242	107 (79.4–135)	186 (158–257)	122 (93.4–145)	178 (128–256)	<0.0001^#^
T. cholesterol (mg/dL)	243	166 (146–192)	183 (145–204)	155 (127–193)	150 (128–182)	0.0069^#^
HDL (mg/dL)	243	43.1 (39.8–49)	37.65 (32.3–42)	44.2 (40.4–52.5)	35.9 (31.2–41.8)	<0.0001^#^
LDL (mg/dL)	243	100 (77.4–116)	102 (70.7–124)	77.8 (54.2–120)	74.6 (56.8–96.1)	0.001^#^
Glucose (mg/dL)	242	88.2 (80.3–94.4)	99 (90.6–127)	93.8 (83.7–103)	105.7 (93–144)	<0.0001^#^
Insulin (mU/L)	242	11.3 (5.44–21)	13.7 (7.89–27.2)	10.9 (4.95–22.9)	12.6 (6.71–30.3)	0.2464 ^#^
HOMA-IR	242	2.25 (1.28–4.63)	3.9 (1.88–7.48)	2.55 (1.1–5.25)	4.65 (2.03–9.75)	0.0083^#^
HbA1c (%)	224	5.7 (5.4–6)	6.2 (5.8–7.3)	5.8 (5.6–6.1)	6.5 (5.9–8.3)	<0.0001^#^
Urea (mg/dL)	242	28.8 (23.5–32.1)	28.7 (23.9–35.7)	33.9 (29.4–40.1)	32.5 (25.9–38.8)	0.0007^#^
Creatinine (mg/dL)	242	0.88 (0.82–1.04)	0.94 (0.82–0.99)	0.95 (0.83–1.1)	0.93 (0.83–1.03)	0.5131^#^
CRP (mg/L)	242	2.03 (1.07–4.76)	2.27 (1.18–6.24)	1.68 (0.8–5.78)	2.76 (1.27–5.36)	0.5146^#^
IL-6 (pg/mL)	243	2.15 (1.5–7.9)	2.6 (1.5–5)	2.7 (1.5–7.6)	3.15 (1.5–6.95)	0.8965^#^
hs-cTnT (ng/L)	240	6.09 (4.12–8.83)	6.24 (4.65–11.7)	6.21 (5.06–12.9)	8.06 (5.79–13.5)	0.1079^#^
Adiponectin (ng/mL)	243	160 (129–191)	51 (39–77.3)	130 (89.5–163)	52.5 (32–117)	<0.0001^#^
FABP4 (ng/mL)	243	5.53 (2.19–10.2)	4.69 (1.64–7.62)	2.93 (0.51–7.62)	0.53 (0.1–4.15)	<0.0001^#^
FSTL1 (ng/mL)	243	31.1 (18.4–45.7)	131 (97.8–158)	33 (25.3–43.8)	108 (64–139)	<0.0001^#^
Irisin (ng/mL)	242	3.89 (2.60–5.78)	7.93 (4.59–10.8)	3.25 (1.66–5.51)	4.97 (2.96–8.06)	<0.0001^#^

SYNTAX score: Calculated based on coronary lesions that produce ≥50% diameter stenosis in vessels with a diameter of ≥1.5 mm. The clinical and demographic characteristics of the participants, along with biochemical analyses, and ELISA measurement results, grouped according to the study categories, are summarized by means ± standard deviations, *n* (%), and medians (25th–75th percentiles) ^*^ ANOVA; ^#^ Kruskal–Wallis test; ^†^ Pearson’s chi-squared test. Group 1: CAD– / MetS–; Group 2: CAD– / MetS+; Group 3: CAD+ / MetS–; Group 4: CAD+ / MetS+. Significant differences in age, BMI, and waist circumference were observed among the four groups; however, no consistent linear trend emerged across all groups, with differences primarily observed between specific group pairs. Abbreviations: BMI: Body mass index; WC: Waist circumference; WHtR: Waist height ratio; HDL: High-density lipoprotein; LDL: Low-density lipoprotein; HOMA-IR: Homeostasis model assessment index; HbA1c: Hemoglobin A1c; DM: Diabetes mellitus; T. cholesterol: Total cholesterol; CRP: C reactive protein; IL-6: Interleukin 6; hs-cTnT: High-sensitive cardiac troponin T; *P*: Significance.

In multivariable logistic regression analyses, circulating levels of adiponectin and FSTL1 remained significantly associated with the presence of metabolic syndrome after adjusting sequentially for age, waist circumference, hypertension, glucose, triglycerides, and HDL levels (Table 3). In contrast, irisin exhibited no significant association with metabolic syndrome in any of the adjusted models.

**Figure 5.**
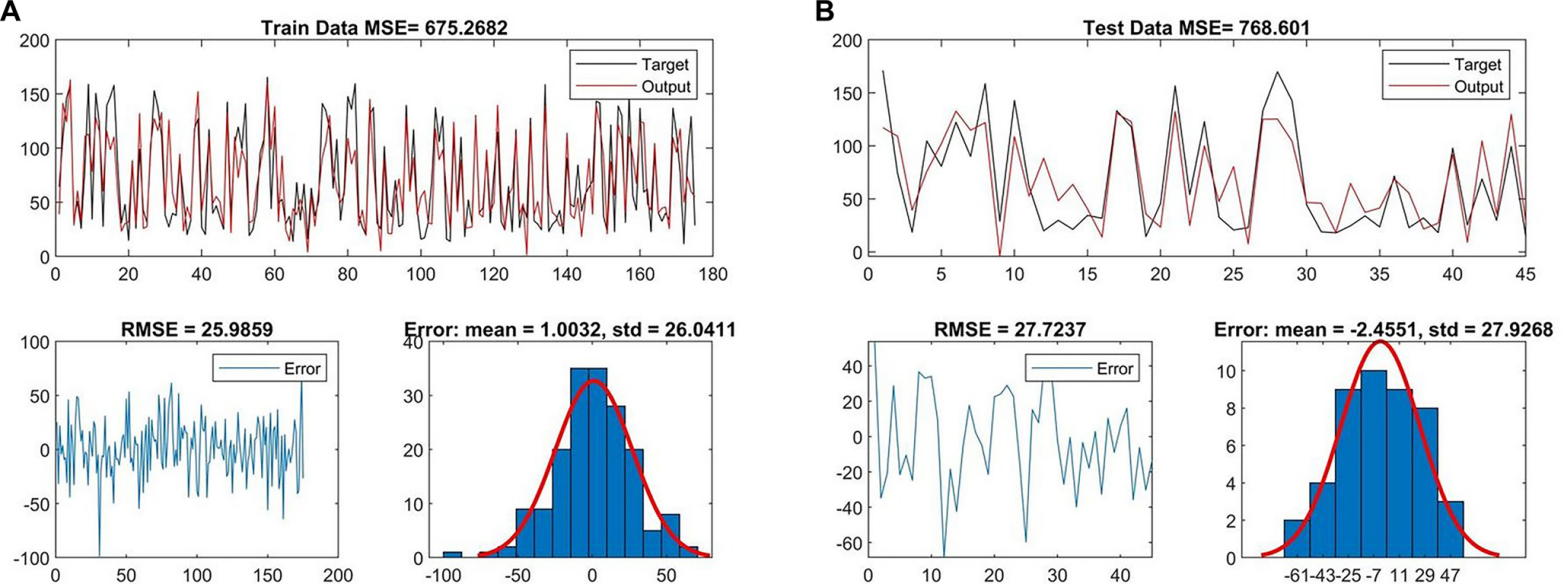
**FSTL1 prediction using the particle swarm optimization-based adaptive neuro-fuzzy inference system method.** (A) Train result; (B) Test result. The model performance for PSO-ANFIS using a single 80–20 train–test split is presented. Abbreviations: RMSE: Root mean squared error; MSE: Mean squared error.

FSTL1 levels in patients were predicted using the PSO-based ANFIS method, incorporating input variables, such as triglycerides, HDL, glucose, HOMA-IR, BMI, adiponectin, irisin, and immature granulocyte counts. To validate the approach, the patient order was randomized prior to analysis. Eighty percent of the patients were assigned to the training set, while the remaining 20% were utilized to evaluate the method’s accuracy. Following the training phase, visual representations of the mean square error (MSE) and root MSE (RMSE), along with their means and SDs, are presented in Figure 5. The performance metrics (RMSE, MAE, R, and R^2^) for all predictive models are detailed in Table 4.

## Discussion

This study investigated the associations of circulating organokines (FABP4, FSTL1, adiponectin, and irisin) with CAD and metabolic syndrome in male patients undergoing elective coronary angiography. Significant variations in the levels of these biomarkers were observed, each demonstrating unique associations relevant to cardiovascular and metabolic diseases.

While literature indicates that FABP4 levels increase in atherosclerotic diseases [19–24], a meta-analysis found that a 1-SD decrease in FABP4 among Type 1 diabetes mellitus patients increased the risk of CAD by 2.4-fold [25]. In our study, FABP4 levels were lower in patients with chronic coronary syndrome. The inflammatory response is known to be significantly heightened in acute coronary syndrome; however, in patients with stable chronic coronary syndrome included in this study, the inflammatory burden is generally lower. Additionally, chronic treatments such as ACE inhibitors, antiplatelet agents, and statins may modulate these levels by affecting the inflammatory response [26, 27]. Our results may be attributed to the higher prevalence of statin use among our CAD patients (57.3%) compared to those without CAD (27.2%). Notably, the association between chronic coronary syndrome and FABP4 persisted after adjusting for dyslipidemia with statin use, suggesting that low FABP4 levels may still serve as a useful predictor in treated patients.

Consistent with previous reports demonstrating reduced circulating irisin levels in patients with stable CAD and inverse associations with disease severity [28, 29], our study also found significantly lower irisin concentrations in the CAD group compared to non-CAD participants (Figure S13). However, this relationship was not maintained in multivariable regression analysis, indicating that the association between irisin and CAD may be confounded by other cardiometabolic factors.

Regarding metabolic syndrome, low adiponectin and high FSTL1 levels were each independently associated with the condition. This finding aligns with prior studies and current biological understanding. Adiponectin, known for its insulin-sensitizing and anti-inflammatory effects, exhibits decreased levels in obesity and metabolic syndrome [30–32]. In our study, adiponectin levels were significantly lower in patients with metabolic syndrome, reinforcing its role as a protective metabolic messenger. Conversely, FSTL1 levels were significantly elevated in individuals with metabolic syndrome, consistent with recent findings identifying FSTL1 as a marker of unhealthy metabolic conditions [33]. The independent association of FSTL1 with metabolic syndrome may reflect inflammation and cardiovascular stress related to metabolic dysfunction. Although FSTL1 is recognized for its protective role in acute conditions and has been associated with CAD severity in other studies [34, 35], we did not observe a significant difference in FSTL1 levels between our CAD and non-CAD groups. This discrepancy may stem from our patient selection (elective, predominantly stable CAD) or the proportion of patients with metabolic syndrome but without CAD (group 2) in our study. The relationship between FSTL1 and heart disease may be context-dependent, with FSTL1 levels increasing primarily in response to metabolic disturbances and inflammation rather than reflecting the extent of atherosclerosis in stable patients. Further research is necessary to elucidate the roles of FSTL1 as both a cardiac and metabolic protein and to determine whether modulating FSTL1 levels influences metabolic or cardiac health.

**Table 2:** Multivariable-adjusted relationships of circulating FABP4 with coronary artery disease

**Model adjust**	**OR**	**95% CI**	***P***
Age	0.857	0.795–0.924	0.000064
Age, dyslipidemia	0.88	0.814–0.952	0.001
Age, dyslipidemia, HT	0.89	0.822–0.965	0.005
Age, dyslipidemia, HT, smoking	0.884	0.810–0.966	0.006
Age, dyslipidemia, HT, smoking, glucose	0.903	0.825–0.987	0.025

CAD classified by SYNTAX score (≥50% stenosis in ≥1.5 mm vessels). Dyslipidemia is defined as total cholesterol ≥200 mg/dL, LDL-C ≥130 mg/dL, triglycerides ≥150 mg/dL, HDL-C <40 mg/dL in men, and/or use of lipid-lowering therapy—Hosmer-Lemeshow test *P* ═ 0.791; Nagelkerke R^2^ ═ 0.361; correct classification rate for CAD status: 76.9%. FABP4 was modeled as a continuous predictor, and an OR less than one indicates that higher values of the predictor are associated with a lower probability of coronary artery disease. Multivariable-adjusted logistic regression analysis showing the independent association of FABP4 levels with the presence of coronary artery disease after sequential adjustment for conventional risk factors. Abbreviations: OR: Odds ratio; CI: Confidence interval; HT: Hypertension; *P*: Significance.

**Table 3:** Multivariable-adjusted relationships of circulating FSTL-1 and adiponectin with metabolic syndrome

	**Adiponectin**			**FSTL1**		
Model adjust	OR	95% CI	*P*	OR	95% CI	*P*
Age	0.975	0.969–0.981	<0.001	1.046	1.035–1.057	<0.001
Age, WC	0.978	0.971–0.984	<0.001	1.045	1.032–1.057	<0.001
Age, WC, HT	0.976	0.968–0.983	<0.001	1.045	1.032–1.058	<0.001
Age, WC, HT, glucose	0.976	0.969–0.984	<0.001	1.044	1.030–1.057	<0.001
Age, WC, HT, glucose, triglyceride	0.978	0.970–0.987	<0.001	1.041	1.027–1.055	<0.001
Age, WC, HT, glucose, triglyceride, HDL	0.979	0.971–0.988	0.000003	1.039	1.025–1.054	<0.001

Odds ratios and 95% confidence intervals were calculated using logistic regression models, progressively adjusting for potential confounders including age, waist circumference, hypertension, glucose levels, triglycerides, and HDL levels. The model incorporating adiponectin achieved a Nagelkerke R^2^ value of 0.722 and accurately classified 87.4% of patients. Conversely, the model including FSTL1 obtained a Nagelkerke R^2^ value of 0.778, with a correct classification rate of 91.5%. Importantly, both adiponectin and FSTL1 remained significantly associated with metabolic syndrome after full adjustment. Abbreviations: HDL: High-density lipoprotein; WC: Waist circumference; HT: Hypertension; *P*: Significance; OR: Odds ratios; CI: Confidence interval.

**Table 4:** Comparison of machine learning model performance metrics in predicting FSTL1 results

**Model**	**Train set**			**Test set**			
	**RMSE**	**MAE**	**R**	**RMSE**	**MAE**	**R**	**R^2^**
Linear regression	32.4	26.5	0.73	31.1±4.23	25.4±4.18	0.64±0.27	0.45±0.32
Random forest	32.5	25.1	0.73	31.4±7.79	25.0±6.04	0.67±0.14	0.47±0.20
k-Nearest neighbors	36.1	27.4	0.65	35.4±8.27	27.7±6.46	0.55±0.23	0.34±0.25
Decision tree	39.2	29.5	0.57	38.1±10	29.2±7.22	0.42±0.33	0.2±0.41
Support vector machine	46.4	37.4	0.22	44.2±9.73	36.2±7.8	0.25±0.2	0.02±0.21
PSO-ANFIS	26	20.2	0.82	16.5±6.34	12.8±4.88	0.91±0.06	0.84±0.1

The test set performance of the models used in FSTL1 level prediction was evaluated using 10-fold cross-validation, and the results are reported as the mean ± standard deviation. Abbreviations: PSO-ANFIS: Particle swarm optimization-enhanced adaptive neuro-fuzzy inference system; RMSE: Root mean square error; MAE: Mean absolute error; R: Correlation coefficient; R^2^: Coefficient of determination.

One remarkable molecule in our study was irisin, a myokine that exhibited elevated levels in patients with metabolic syndrome. The role of irisin in metabolic diseases remains contentious. Numerous studies align with our findings [36–38], indicating higher irisin levels in individuals with Type 2 diabetes, which have been associated with endothelial activation [39]. These increases are typically interpreted as a compensatory response. In this context, higher irisin levels may help prevent weight gain and insulin resistance by enhancing energy expenditure and promoting glucose uptake in muscle [40]. Our findings support this perspective, suggesting an active interplay between muscle and adipose tissues in metabolic syndrome. However, some studies contradict our results, reporting either lower or stable irisin levels in obesity and metabolic disorders [41, 42]. Variability among patient groups, including differences in exercise habits, muscle mass, disease status, and the lack of analytical standardization, may contribute to these discrepancies [43]. Although irisin has been identified in other research as a predictor of CAD, metabolic syndrome, and adverse cardiovascular outcomes, it did not emerge as an independent predictor in our analysis. Overall, our findings and the divergent results in the literature indicate that the role of irisin in metabolic syndrome is complex. While it may serve as a flexible response mechanism, its utility as a standalone biomarker is limited due to variability. Standardizing irisin measurements and exploring various subgroups, such as active vs inactive individuals, necessitates further investigation to clarify when irisin serves as an optimal indicator of metabolic risk. Elevated irisin levels in the metabolic syndrome cohort reinforce the notion of intercommunication among signals from muscle, fat, and heart in this context.

The multimarker approach utilizing adiponectin, FSTL1, and irisin demonstrates high diagnostic performance for metabolic syndrome (AUC = 0.92). High sensitivity and specificity values were observed through the joint evaluation of interactive signals across different organ systems. The integration of various metabolic indicators has led to a more comprehensive assessment of metabolic risk. Nonetheless, challenges such as assay costs, test accessibility, and lack of standardization must be addressed to facilitate the incorporation of this approach into routine clinical practice.

To the authors’ knowledge, this study presents the first AI-based PSO-ANFIS model for estimation within a clinical dataset, achieving an R^2^ value of 0.84, surpassing other methods. The success of our model underscores the interconnectedness of metabolic messengers, supporting the notion that FSTL1 reflects signals from multiple metabolic pathways and responds to overall metabolic stress. Additionally, our results demonstrate how computational intelligence can enhance traditional biostatistical methods. While classical approaches primarily identify linear relationships, the ANFIS framework captures complex, non-linear interactions that may otherwise remain undetected.

However, the current model should be viewed as proof of concept. Although it accurately predicts FSTL1 levels, its clinical application remains experimental. In situations where direct measurement of FSTL1 is impractical or costly, the model may provide valuable estimates or assist in monitoring responses to therapeutic or lifestyle interventions. Further validation in larger, independent cohorts is necessary before any clinical application can be considered.

From a clinical perspective, assessing a broader panel of organokines could enhance risk stratification for CAD and metabolic syndrome, aiding clinicians in identifying individuals who may benefit from more intensive management of modifiable risk factors. The strong associations observed between FABP4 and FSTL1 and cardiometabolic disease suggest that these molecules could serve as future therapeutic targets. For instance, pharmacological inhibition of FABP4 is already being investigated for metabolic disorders [44, 45], while interventions enhancing adiponectin signaling, through PPAR-γ agonists [46] or lifestyle modifications, are known to yield metabolic improvements. AMP-activated protein kinase (AMPK), which acts through adiponectin and SGLT-2 inhibitors, is a critical mediator linking these organokines to cardiometabolic protection and may serve as a therapeutic target [47, 48].

Even though specific therapies targeting FSTL1 or irisin do not yet exist, a deeper understanding of their functions could unlock new therapeutic possibilities. Modulating FSTL1 activity may have beneficial effects on inflammatory or fibrotic pathways in the heart and vasculature, while the metabolic benefits of exercise may be mimicked through irisin signaling.

Consistent with studies linking adipokines from the C1q/TNF-related protein (CTRP) family to subclinical atherosclerosis, such as increased carotid intima-media thickness, our findings underscore the relevance of adipokine-related mechanisms to vascular dysfunction by demonstrating that FABP4 contributes to the association with CAD [49].

In summary, our findings encourage ongoing investigation into whether modulating organokine levels could ultimately reduce cardiovascular events or enhance metabolic outcomes. Due to the cross-sectional design of this study, causal relationships between biomarkers and clinical outcomes cannot be established. The cohort included only patients undergoing coronary angiography and therefore consisted of individuals with low to moderate risk of CAD. Antidiabetic, antihypertensive, and lipid-lowering therapies may influence the biomarker levels we measured, and a limitation is that their effects could not be evaluated alongside their dosages in this study. Given the relatively small sample size, the results of the AI model should be interpreted with caution and validated in external datasets.

A primary limitation of this research is that the cohort was restricted to middle-aged and elderly male patients. This selection was intentional to minimize variability arising from sex-specific biological and hormonal influences. It is well established that circulating concentrations of adiponectin, FABP4, irisin, and FSTL1 vary with sex hormones, menopausal status, fat distribution, and skeletal muscle mass. For example, adiponectin levels are typically higher in women than in men [50]. FABP4 concentrations and their relationships with cardiometabolic risk factors may also differ by gender. Likewise, irisin secretion and its metabolic effects are influenced by muscle mass and physical activity, both of which exhibit gender-dependent variations. Future research should consider the limitations of our study, particularly the male-only cohort derived from elective angiography, which restricts generalizability. Longitudinal or interventional studies encompassing a broader patient risk spectrum are required to validate our findings.

## Conclusion

This research highlights FABP4 and FSTL1 as significant biomarkers associated with CAD and metabolic syndrome, respectively, while reaffirming the roles of adiponectin and irisin in metabolic regulation. The concurrent evaluation of these organokines significantly enhances the diagnostic accuracy for metabolic syndrome and encourages the adoption of multi-marker approaches in clinical practice. Furthermore, AI-based predictive models underscore the potential of innovative analytical methods in biomarker research, facilitating improved personalized medical strategies for cardiometabolic diseases.

## Supplemental data

Supplemental data are available at the following link: https://www.bjbms.org/ojs/index.php/bjbms/article/view/13188/4036.

## Data Availability

Upon reasonable request, the corresponding author will provide the study’s data.
